# Survival time and prognostic factors of patients with initial noncurative colorectal liver metastases

**DOI:** 10.1097/MD.0000000000008831

**Published:** 2017-12-22

**Authors:** Qizhi Liu, Liqiang Hao, Zheng Lou, Xianhua Gao, Haifeng Gong, Yonggang Hong, Chuangang Fu, Wei Zhang

**Affiliations:** a Department of Colorectal Surgery, Changhai Hospital, Second Military Medical University; b Department of Colorectal Surgery, Shanghai East Hospital, Tongji University School of Medicine, Shanghai, China.

**Keywords:** colorectal liver metastasis, liver resection, primary tumor resection

## Abstract

The true survival benefit of different curative strategies involving type of operative procedure and timing for patients with initial noncurative colorectal liver metastases remains uncertain. The goal of this study was to examine the effect of primary tumor resection on patients’ survival and to clarify the predictive factors related to overall survival (OS).

This was a retrospective study that included 219 patients with initial noncurative colorectal liver metastases without extrahepatic disease. The clinicopathological characteristics of patients and their survival were examined. Survival analysis was performed using the Kaplan–Meier method. All variables associated with *P* <.05 in univariate analysis were included in multivariate analysis using a Cox proportional-hazard regression model.

The 1-, 3-, 5-year OS rates of patients with simultaneous liver resection were 79.1%, 39.1%, and 28.4%, respectively, and those of patients with staged liver resection were 83.3%, 46.7%, and 36.8%, respectively (*P* = .380). The 1-, 3-, 5-year OS rates of patients with primary tumor resection were 57.0%, 18.2%, and 12.3%, respectively, while for the patients without primary tumor resection were 38.9%, 5.6%, and 0%, respectively (*P* = .012). Independent prognostic factors for OS were carbohydrate antigen19–9, primary tumor resection, tumor differentiation, and adjuvant chemotherapy.

No difference in OS was observed between simultaneous liver resection and staged liver resection, while primary tumor resection was beneficial to noncurative colorectal liver metastases.

## 1 Introduction

Colorectal cancer (CRC) is among the top 3 most common cancers and one of the leading causes of death worldwide.^[1]^ Almost 1 quarter of all CRC patients present with synchronous liver metastases at the time of initial diagnosis, while around 2 to 3 quarters of patients have recurrence within 3 years after surgical removal of the primary tumor.^[2]^

Though most patients with colorectal liver metastases (CRLM) have a poor prognosis, some patients can still benefit from radical surgery and possibly even avoid recurrence.^[3,4]^ However, for patients with incurable CRLM disease, the optimal management of the primary tumor remains an unanswered clinical question. Several retrospective studies and meta-analyses have demonstrated that patients with primary tumor resection had improved survival outcomes, with a 3 to 6 month improvement in overall survival (OS).^[5–7]^ However, the data have not been entirely consistent on the basis of published studies.^[8,9]^

Although various prognostic factors that determine the outcome of CRLM patients after liver resection have been identified, including tumor-free surgery margin, TNM stage, number of metastases, and so on,^[10]^ there have been no large-cohort reports on the long-term survival of metastatic patients in China. Thus, in this study, we retrospectively assessed possible prognostic factors for synchronous CRLM in a cohort of Chinese patients.

## 2 Patients and methods

### 2.1 Patient selection

This study included 219 initial noncurative CRLM patients between January 2004 and December 2014 at Changhai Hospital, Second Military Medical University, Shanghai, China. The clinicopathological characteristics of the patients, their primary tumor, and its metastases were retrospectively reviewed, including gender, age, intestinal obstruction, carcinoembryonic antigen (CEA) serum levels, carbohydrate antigen19-9 (CA19-9) serum levels, tumor shape, surgical treatment, microscopically negative/positive surgical margin (R0/R1) resection, T stage, N stage, and tumor differentiation. Postoperative adjuvant chemotherapy after surgery, and survival time were recorded as well. The tumor stage was determined according to the seventh edition of the TNM classification system of the American Joint Committee on Cancer (AJCC) for primary CRC.

All patients underwent computed tomography (CT) of the preoperative chest and abdominopelvic area with magnetic resonance imaging (MRI) of the liver to detect the presence of metastases. The resected specimens (primary tumor or liver metastasis) were pathohistologically proven as adenocarcinoma. Neoadjuvant chemotherapy was administrated to all patients. As first-line chemotherapy, oxaliplatin-based and irinotecan regimens were administered. In addition, anti-VEGF therapy was combined with systemic chemotherapy. If patient had a wild-type RAS status, anti-EGFR therapy was combined with systemic chemotherapy. Patients were followed using CT/MRI scanning and resectability was assessed every 2 or 3 months. Chemotherapy was continued until primary tumor or liver metastasis resection could be performed, or until the patient could no longer receive chemotherapy due to tumor progression, or adverse reaction to treatment. OS was defined as the time from the date of surgery to the confirmed death date for dead patients or from the date of surgery to the date of the last follow-up for surviving patients.

The study was approved by the medical ethics board of Changhai Hospital (the ethical approval number of the study is CHEC2015-146, and informed consent was obtained from all patients.

### 2.2 Liver resection

Primary hepatectomy was performed if technically possible. Decision on possible resection was performed based on the size of the remnant liver volume (expected at least 30% functional liver after hepatectomy).

### 2.3 Postoperative follow-up of patients

In our follow-up study, data from all patients were censored from the date of surgery to the date of the last follow-up visit (December 31, 2015) or death. Patients were evaluated every 3 months during the first postoperative year, every 6 months in the second year, and once annually thereafter. During the follow-up period, the date of death and the cause of death were recorded. All patients were monitored by detection of CEA and CA19-9 levels, colonoscopy, liver MRI, and chest and abdominopelvic CT.

### 2.4 Statistical analysis

Statistical Package for Social Science version 17.0 (SPSS, Chicago, IL) software was used for statistical analysis. The relationship between potential prognostic factors was evaluated using Pearson's χ^2^ test or a χ^2^ 2-tailed Fisher exact test, as indicated, followed by multivariate analysis using Cox regression. The results were considered significant with *P* <.05 (2-sided). Survival analyses were performed using the Kaplan–Meier method, and the log-rank test was applied to determine the influence of variables on OS. All variables associated with survival that had *P* <.05 in univariate analysis were entered into multivariate analysis using a Cox proportional hazard regression model.

## 3 Results

### 3.1 Clinicopathological characteristics of patients

**Figure 1:**
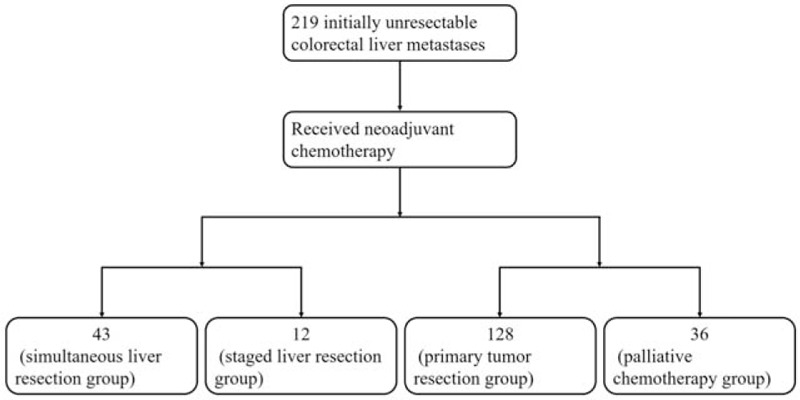
Flow diagram of the 219 patients with initial noncurative colorectal liver metastases. After neoadjuvant chemotherapy, 55 patients were converted to be resectable. Among them, 43 patients underwent simultaneous primary tumor resection and hepatectomy (simultaneous liver resection group), 12 patients underwent primary tumor resection first followed by hepatectomy (staged liver resection group). Among the 164 patients with unresectable livers after chemotherapy, primary tumor resection was applied to 128 patients (primary tumor resection group), the remaining 36 patients underwent adjuvant chemotherapy (palliative chemotherapy group).

A total of 219 patients with initial noncurative CRLM treated were included in the study. A flow diagram of patient stratification is presented in Figure 1. After neoadjuvant chemotherapy, 55 initially unresectable patients at the time of diagnosis were converted to resectable. Among these patients, 43 underwent simultaneous primary tumor resection and hepatectomy (simultaneous liver resection group). Twelve patients underwent primary tumor resection first followed by hepatectomy (staged liver resection group). The remaining 164 patients were still unresectable even after chemotherapy. Among the 164 patients with unresectable livers, primary tumor resection was applied to 128 patients (primary tumor resection group), the remaining 36 patients underwent adjuvant chemotherapy (palliative chemotherapy group).

### 3.2 Survival analysis

**Figure 2:**
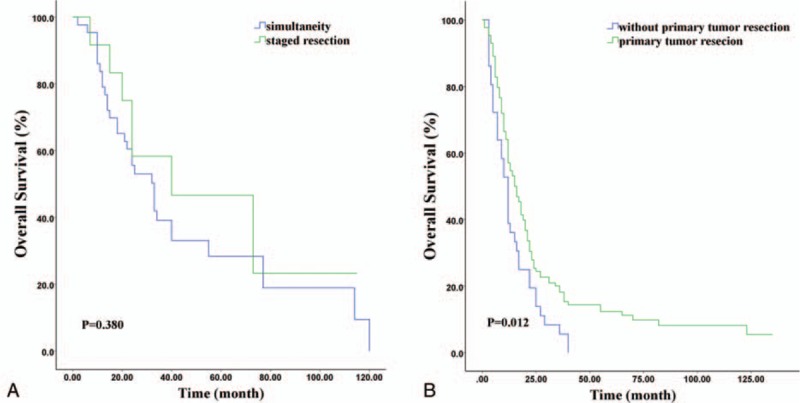
Prognostic significance assessed using Kaplan–Meier survival estimates and log-rank tests. (A) Kaplan–Meier survival curves showing no difference between simultaneous and staged resection (*P* = .380, log-rank test). B. Kaplan–Meier survival curves showing a significantly prolonged OS among patients with primary tumor resection compared with patients without primary tumor resection (*P* = .012, log-rank test). OS = overall survival.

The median follow-up period for patients included in this study was 16 months (range 1–135 months). The 1-, 3-, and 5-year OS rates of the 219 patients were 59.8%, 24.0%, and 15.5%, respectively, with a median of 16 months. For 55 liver resection patients, the 1-, 3-, and 5-year OS rates of patients with simultaneous liver resection were 79.1%, 39.1%, and 28.4%, respectively, and those of patients with staged liver resection were 83.3%, 46.7%, and 36.8%, respectively (*P* = .380) (Fig. 2A). For remaining 164 unresectable liver metastases after chemotherapy, the 1-, 3-, and 5-year OS rates of patients with primary tumor resection were 57.0%, 18.2%, and 12.3%, respectively, while for the patients without primary tumor resection were 38.9%, 5.6%, and 0%, respectively (*P* = .012) (Fig. 2B).

### 3.3 Univariate and multivariate analysis of potential survival predicting factors

In univariate analysis, gender, age, intestinal obstruction, CEA level, CA19-9 level, tumor shape, primary tumor resection, surgical treatment, R0 resection, T stage, N stage, tumor differentiation, and postoperative adjuvant chemotherapy were included for survival analysis. Based on analyses, prognostic factors related to worse OS in univariate analysis were age ≥60, CEA level ≥5 ng/mL, CA19-9 level ≥37 U/mL, without primary tumor resection, R1 resection, N1/N2 stage, poor tumor differentiation, and no adjuvant chemotherapy (Table 1).

**Table 1:**
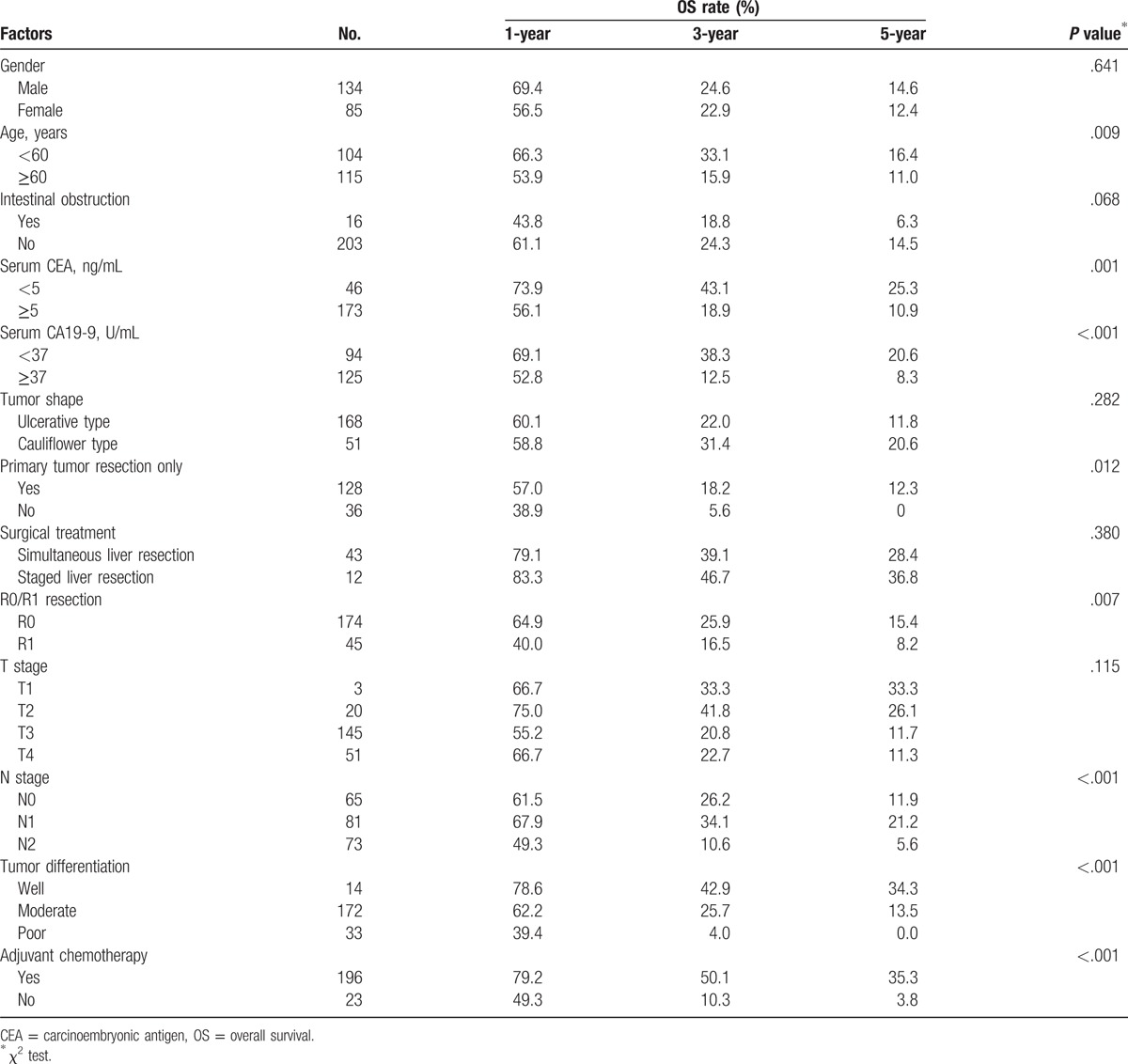
Univariate analysis of factors associated with 1-, 3-, 5-year OS.

Those parameters that were significant predictors for OS were then included in a multivariate Cox proportional hazards model for analysis. The results were as follows: CA19-9 (hazard ratio, [HR] 1.389; 95% confidence interval [CI], 1.009–1.912; *P* = .044), primary tumor resection (HR, 0.598; CI, 0.393–0.909; *P* *=* .016), tumor differentiation (HR, 1.512; CI, 1.091–2.098; *P* = .013), and adjuvant chemotherapy (HR, 0.408; CI, 0.281–0.591; *P* <.001) were independent prognostic factors for OS (Table 2). Further, CA19-9 and tumor differentiation were risk factors for CRLM patients, and primary tumor resection and adjuvant chemotherapy were protective factors for CRLM patients.

**Table 2:**
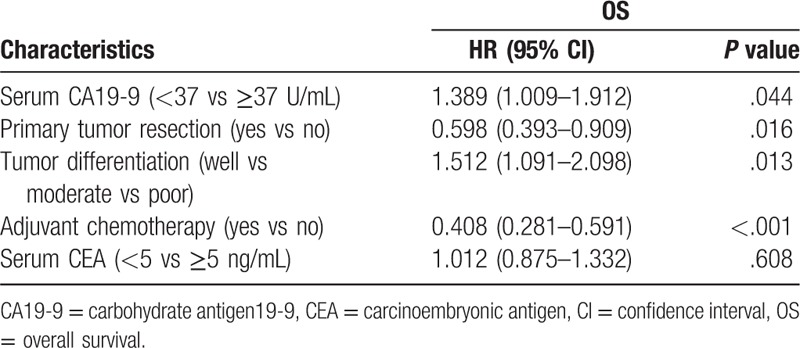
Cox's multivariate analysis for OS.

## 4 Discussion

Liver metastases are the first site of metastasis in most CRC patients. Liver resection is the standard line of treatment of CRLM patients, however the optimal timing for performing this procedure is still not clearly defined.^[11]^ Traditionally surgical removal of the colorectal tumor was performed first. Afterwards, patients were treated with adjuvant chemotherapy, and finally a staged liver resection was performed. By contrast, the most recent data are in favor of the simultaneous primary colorectal tumor as well as liver resections due to its proven safety.^[12]^ Silberhumer et al^[13]^ reported that patients with simultaneous liver and colorectal resections have similar long-term cancer outcomes compared to those which were submitted to staged resection. Our data also showed similar results for simultaneous and staged resections for Chinese patients. Thus, based on these findings, it can be concluded that the choice of therapeutic strategy for each patient will depend on the physical condition.

In this study, for patients diagnosed with unresectable metastases after neoadjuvant chemotherapy, prior resection of the primary tumor was independently associated with a better OS. In a large homogeneous population study, Venderbosch et al^[16]^ assessed the influence of primary tumor resection in patients included in the CAIRO-1^[14]^ and CAIRO-2^[15]^ phase III study and in both studies primary tumor resection was independently associated with better OS.^[16]^ The mechanisms concerning the positive effect of the resection of the primary tumor remain elusive. This is probably due to the fact that primary tumor resection have lower metastatic burden that may significantly influence survival.^[6]^ Nevertheless, it has also been reported that liver tissue adjacent to metastases provides better environment for metastatic tumor growth when primary tumor is not resected.^[17]^

Many clinical prognostic factors have been identified in patients with CRLM, including poorly differentiated tumors, higher number and size of the metastases, tumor stage, presence of extrahepatic metastasis, elevated CEA levels, and positive nodal status,^[18]^ however, there is still not final agreement on which of these factors has the best prognostic value.

Relapse within the liver is very common in the first 2 years after the operation.^[19]^ The role of systemic adjuvant chemotherapy after metastasectomy to decrease this recurrence has been reported in several studies. Mitry et al^[20]^ for instance demonstrated benefits of adjuvant 5-FU based chemotherapy in both disease free survival (DFS) and OS. In a study by Portier et al^[21]^ patients receiving postoperative systemic FU plus LV had significantly better survival than those receiving surgery alone. In this study, we found that adjuvant therapy after metastasectomy improved OS and was an independent prognostic factor for OS.

For many patients, CEA can be a useful marker in monitoring recurrence, as well as assessing the response to adjuvant chemotherapy. For instance, Mann et al^[22]^ reported that preoperative CEA levels (CEA levels <200 ng/mL: 48.9% vs >200 ng/mL: 0.0%) were correlated with 5-year survival. Other studies have also demonstrated that preoperative CEA levels >200 ng/mL were an independent factor for poor OS and DFS.^[23,24]^ In contrast with these studies, other researchers have reported that preoperative CEA level was not a significant predictor of survival or recurrence after CRC hepatectomy.^[25,26]^ In our study, multivariate Cox analysis showed that preoperative CEA was not an independent significant factor for the OS factor.

Serum CA19-9 levels have also been reported as an independent prognostic predictor of survival in patients with unresectable CRLM.^[27]^ Our results confirmed this, since CA19-9 was an independent significant factor for OS. Nevertheless, in other studies CA19-9 was not found to be a significant factor for OS.^[28]^ The reason for these differences in results may be the different cut-off values used for CEA and CA19-9.

Our study has several limitations. First, this study includes the relatively small number of patients without negative control of CRLM patients without any surgery since many of them were excluded due to the lack of information regarding their clinicopathological features. The patients’ treatments were different so that the baseline levels were inconsistency, so that it may produced some bias to relative risk factors about CRLM. Second, this was a single-centered study, and this might result in the selection bias. Third, other prognostic variables (RAS and BRAF mutations) were not available for this study. Thomsen et al^[29]^ reported that RAS and BRAF mutations in serum combined with pMMR were strong independent prognostic factors in colon cancer patients. In the present study, the 5-year survival rate in without liver resection group was 9.7%. This survival rate was longer than the previous reports (4%).^[30]^ These findings might possibly be explained by the resection of the primary tumor.

In conclusion, primary tumor resection in noncurative colorectal liver metastases results in improved patients’ survival, particularly in patients with good general condition. The clinicopathological prognostic factors identified may help identify high-risk patients and be useful in the consideration of further treatments.

## Acknowledgment

The authors thank Medjaden Bioscience Limited for their careful polish of the article.
